# Supplementary material for: Machine learning algorithm for ventilator mode selection, pressure and volume control
Source: PLoS One. 2024 Mar 13;19(3):e0299653. doi: 10.1371/journal.pone.0299653 (PMC10936794; doi:10.1371/journal.pone.0299653)
Supplement: Attachment — Submitted filename: Response to Reviewers.pdf [file pone.0299653.s002.pdf]

Title: Classifier Machine Learning Algorithm for Ventilator Mode Selection, Pressure and Volume Control

Manuscript ID: PLOS ONE - PONE-D-23-25069

Reviewer	S.No	Comment	Response
**1**	1.	Author need to synchronize the usage of the classifier terms in this research similar to the term written in Fig 3.	We are grateful for your recognition of our research article. Your review comments are highly esteemed as they allow us to enhance the quality of our work.<br>In the revised article, as per the suggestions, terms are synchronized and addressed everywhere. Further, the terms are abbreviated and given in the abbreviation section.<br>**(See Page 10, highlighted part, Lines 345 to 348.)**
	2.	Author need to further elaborate the steps/activities involves in the classifier framework (fig 4) and relate with the discussion in line 205.	Thank you for your valuable suggestions. In the updated article, we have added the activities involved in every phase of the proposed framework with the respective correlation of each step.<br>**(See Page 7, highlighted sentences, Lines 210 to 231.)**
	3.	Please modify the section number in the summarization of introduction (end paragraph of introduction section) accordingly.	We greatly appreciate your suggestions. We have updated the Introduction section number.<br>**(See Page 1, Section title.)**
	4.	Please write/refer to the exact figure no and table no. and not general statement of figure and number (e.g line 341)	As per the suggestion, we have updated the table and figure calling in the revised article.<br>**(See Pages 10 and 11, Lines 375-378.)**

**2**	1.	Hyperparameters need to be mentioned for ML classifiers.	Thanks for your kind suggestions, and your review comments are highly esteemed as they allow us to enhance the quality of our work.<br>In the updated article, the hyperparameters used for the ML classifiers are updated and given in the results and discussion section.<br>**(See Page 10, highlighted sentences, Lines 339 to 345.)**
	2.	Whether 5 fold or 10 fold validation performed?	In our research study, we have used 5 folds for performance validation.
	3.	Image quality need to be improved	Thanks for your kind suggestions. We have generated all our results in EPS format. However, the submission portal only allows PDF or TIFF, so the conversion caused this issue. However, we use PDF format in the final upload to avoid this issue.
	4.	Contributions should have crisp points.	Thanks for your kind suggestions. We have updated the article's contributions and significance in this updated version.<br>**(See Page 4, highlighted sentences, Lines 104 to 123.)**
	5.	Fig 5 is general...hence can be removed.	As per the suggestions, we have removed Fig 5.
	6.	Discussion of proposed work results with existing literature works needs to be improved.	The proposed ILCPID controller technique is compared with the existing PID controller. Also, the classifiers are compared with different methods like decision trees, optimizable, naive Bayes, nearest neighbour, ensemble classifier, and neural network. In addition, we have further improved the existing literature works.

**3**	1.	The article is well written in terms of problem specifications, modeling, and results according to the different machine learning algorithms. The article is also suitable in terms of scientific contributions.	Thank you for acknowledging our research article. We greatly appreciate your review comments and believe they will allow us to improve the quality of our work. Your feedback is invaluable in helping us to refine and enhance our research.
	2.	Only thing which I have noticed to be rectified in Figures, 12, 13, 17 and 18 for term 'Decision Trees'. Which has to be corrected. Else is fine.	Thanks for your kind suggestions. In Figures 12, 13, 17 and 18, in order to avoid the repetition of the word “trees” in all the ML classifiers, we have kept them on the vertical axis for better readability. However, we have addressed and rewritten this in the results and discussion section. **(See Page 10, highlighted part, Lines 345 to 348.)**
